# Biochemical Characterization of a Human Septin Octamer

**DOI:** 10.3389/fcell.2022.771388

**Published:** 2022-03-03

**Authors:** Martin Fischer, Dominik Frank, Reinhild Rösler, Nils Johnsson, Thomas Gronemeyer

**Affiliations:** ^1^ Institute of Molecular Genetics and Cell Biology, Ulm University, Ulm, Germany; ^2^ Core Unit Mass Spectrometry and Proteomics, Ulm University, Ulm, Germany

**Keywords:** septins, septin octamer, GTPases, nucleotide uptake, actin polymerization

## Abstract

Septins are part of the cytoskeleton and polymerize into non-polar filaments of heteromeric hexamers or octamers. They belong to the class of P-loop GTPases but the roles of GTP binding and hydrolysis on filament formation and dynamics are not well understood. The basic human septin building block is the septin rod, a hetero-octamer composed of SEPT2, SEPT6, SEPT7, and SEPT9 with a stoichiometry of 2:2:2:2 (2-6-7-9-9-7-6-2). Septin rods polymerize by end-to-end and lateral joining into linear filaments and higher ordered structures such as rings, sheets, and gauzes. We purified a recombinant human septin octamer from *E. coli* for *in vitro* experimentation that is able to polymerize into filaments. We could show that the C-terminal region of the central SEPT9 subunit contributes to filament formation and that the human septin rod decreases the rate of *in vitro* actin polymerization. We provide further first kinetic data on the nucleotide uptake- and exchange properties of human hexameric and octameric septin rods. We could show that nucleotide uptake prior to hydrolysis is a dynamic process and that a bound nucleotide is exchangeable. However, the hydrolyzed γ-phosphate is not released from the native protein complex. We consequently propose that GTP hydrolysis in human septins does not follow the typical mechanism known from other small GTPases.

## 1 Introduction

Septins were discovered in yeast in 1971 (Hartwell 1971) and are part of the cytoskeleton (Mostowy and Cossart 2012). They form non-polar filaments that unlike actin filaments and microtubules do not serve as railroad tracks for motor proteins. Being rather neglected for several years after their discovery, research on the septins gained a growing attention over the past few years (Menon and Gaestel, 2017). Initially labeled as passive scaffold proteins, septins were later discovered to actively participate in many dynamic intracellular processes. Septins regulate the organization of the cytoskeleton, vesicle transport and fusion, chromosome alignment and segregation, and cytokinesis (Surka et al., 2002; Estey et al., 2010; Bowen et al., 2011; Füchtbauer et al., 2011; Tokhtaeva et al., 2015).

The septin subunit SEPT9 was reported to bundle actin and microtubules (Bai et al., 2013; Dolat et al., 2014; Mavrakis et al., 2014; Smith et al., 2015).

The mammalian genome encodes thirteen different septins (SEPT1-SEPT12, SEPT14) (Russell and Hall, 2011), of which SEPT2, SEPT7 and SEPT9 are nearly ubiquitously expressed, while SEPT1, SEPT3, SEPT12, and SEPT14 appear tissue-specific. All septin subunits can be sorted into four subgroups based on sequence homology, namely the SEPT2, SEPT3, SEPT6 and SEPT7 subgroup (Kinoshita 2003; Pan et al., 2007).

The basic septin building block in mammalian cells is a hetero-octamer composed of the SEPT2, SEPT6, SEPT7, and SEPT9 at a stoichiometry of 2:2:2:2 (2-6-7-9-9-7-6-2) (Soroor et al., 2021). This building block is capable of polymerizing by end-to-end and lateral joining into higher ordered structures such as rings, filaments, and gauzes. Assembly into these higher ordered structures is achieved by alternating interactions between the G domains (G interface) or between the N- and C-termini (NC interface), respectively (Bertin et al., 2008).

All septins share a central GTP binding domain (short G domain) that is flanked by variable C- and N-terminal extensions (Sirajuddin et al., 2007) and contains a septin unique element including a β-meander and two further helices. The G domain is conserved among different species and is supposed to be loaded either with GTP or GDP. The available crystal structures of human and yeast septins (Sirajuddin et al., 2007; Sirajuddin et al., 2009; Zent et al., 2011; Brausemann et al., 2016; Valadares et al., 2017; Castro et al., 2020; Rosa et al., 2020) confirm that the G domain contains all structural features of small GTPases like the P-loop and the distinct Switch1 and Switch2 loops including the invariant DXXG motif. GTP binding and hydrolysis was confirmed for human septins. Standard assays aiming at detecting GTP hydrolysis in small GTPases employ usually loading of the GTPase with a GTP labeled at its γ-phosphate and subsequent detection of the released γ-phosphate (Frech et al., 1990) or detection of GTP decrease and GDP increase upon hydrolysis by HPLC (Tucker et al., 1986). However, detection of GTPase hydrolysis products in human septin subunits was so far only achieved after denaturation of the protein (Sirajuddin et al., 2009; Castro et al., 2020). GTP uptake and hydrolysis of entire rods composed of human septins was not yet examined. However, these experiments were conducted for entire rods from yeast, leading to partly contradictory results (Vrabioiu et al., 2004; Farkasovsky et al., 2005; Baur et al., 2018).

Only one of the available septin crystal structures, the human SEPT2/6/7 complex (Sirajuddin et al., 2007), shows a trimeric complex composed of more than two different subunits in the asymmetric unit. A recently presented hexameric structure of the same complex was built based on cryo-EM data (Mendonça et al., 2021). All other human septin structures contain various compositions of maximal two different subunits. Octameric rods for *in vitro* experimentation were only presented recently (DeRose et al., 2020; Iv et al., 2021) and now in this work. We present here a first biochemical characterization of the nucleotide binding- and hydrolysis properties of purified human octameric septin rods and measure the influence of septin complexes on actin filament assembly in real time.

## 2 Materials and Methods

### 2.1 Molecular Cloning, Protein Purification- and Analysis

The ORFs of the isoforms i1 of SEPT2 and SEPT9 (or SEPT91-568) as well as SEPT7 and SEPT6 were cloned into two compatible bicistronic plasmids. SEPT6 was constructed with a N-terminal 6His-tag for IMAC purification while the other subunits were untagged.

Protein expression of hexameric and octameric rods was performed in the *E. coli* strain BL21DE3 in SB medium at 18°C. Protein purification from the crude extract was performed by immobilized metal affinity chromatography (IMAC) and subsequent size exclusion chromatography (SEC).

Integrity of the purified complex was determined by density gradient centrifugation and MS analysis. Western blot analysis was performed using an anti-SEPT9 antibody (mouse, clone 2C6, Sigma Aldrich). Detailed protocols on the cloning-expression and purification procedures as well as on the analytical assays can be found in the supporting methods.

### 2.2 Filament Formation Assays

Septin filament formation was performed as described elsewhere for yeast septins (Renz et al., 2013). Briefly, septin preparations were adjusted to 2 µM and cleared from aggregates by centrifugation. Filament formation was induced by dialysis into 50 mM Tris pH 8.0, followed by incubation at 4°C overnight and subsequent centrifugation at 100.000 xg. The pellet was resuspended in 50 mM Tris pH 8.0 and either subjected to SDS-PAGE or to negative staining for electron microscopy. Therefore, the protein solution was spotted onto a carbon coated TEM grid and negative stained with uranyl acetate using standard procedures. EM imaging was performed on a JEM 1400 TEM instrument (Jeol) at 120 kV acceleration voltage. The camera resolution was 2000 x 2000 px.

### 2.3 Nucleotide Uptake- and Hydrolysis Assays

The nucleotide content of the rod preparations was determined by analytical anion exchange chromatography. Briefly, purified protein from the SEC was dialyzed into buffer without salt and subsequently heat denatured to release bound nucleotide. The denatured protein was pelleted and the supernatant was loaded onto a MonoQ HR 5/5 column (Amersham Pharmacia Biotech). The nucleotides were eluted by a linear salt gradient and detected at 254 nm. GTP and GDP were used to determine the elution profiles of the pure compounds.

Uptake of [γ^32^P]-GTP to hexameric and octameric rods and isolated SEPT9 was performed as described elsewhere for yeast septins (Baur et al., 2018). Briefly, septin preparations were incubated with [γ^32^P]-GTP in exchange buffer containing 5 mM EDTA. For hydrolysis, the exchange reaction was transferred into hydrolysis buffer containing MgCl_2_. Radioactivity retained in the native septin proteins during uptake or during hydrolysis was detected with a filter binding assay.

Further details of the procedure and the subsequent data processing are outlined in the supporting methods.

### 2.4 Actin Polymerization Assays

Pyrene-labeled G-actin (shortly pyrene actin) was prepared from rabbit muscle acetone powder as described elsewhere (Cooper et al., 1983) with some modifications outlined in the supporting methods. Pyrene actin fluorescence increases upon actin polymerization and can be recorded in actin polymerization buffer at an excitation of 365 nm and emission of 407 nm. Details on the pyrene actin preparation, the assay protocol and data processing are outlined in the supporting methods.

The unprocessed raw data are shown in Supplementary Figure S4.

## 3 Results

### 3.1 Construction and Purification of Human Hexameric- and Octameric Septin Rods

We expressed the subunits of the human octameric and hexameric septin rods in *E.coli* from two compatible bicistronic plasmids. SEPT6 and SEPT7 were expressed from one plasmid and SEPT2 (and SEPT9 for octameric rods) from the second plasmid as in a previous study (Sheffield et al., 2003). Only SEPT6 carried a hexa-histidine tag for purification by immobilized metal affinity chromatography (IMAC). The enriched fractions were subsequently subjected to size exclusion chromatography (SEC). Complexes eluted in a shoulder in the second half of the main peak (the first half representing the void peak of the column) and in a minor peak directly adjacent to the main peak (Supplementary Figure S1A).

**FIGURE 1:**
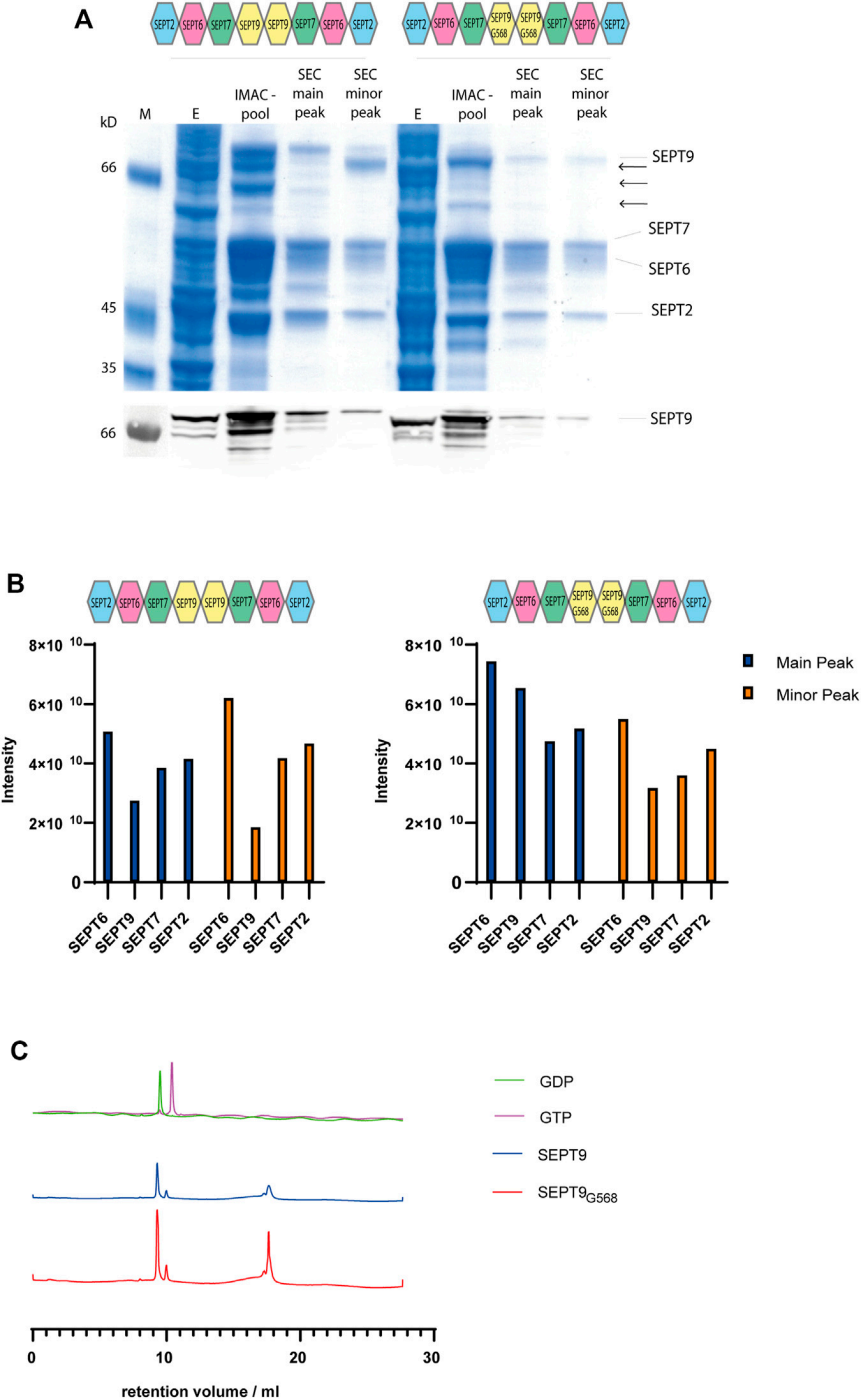
Purification of octameric septin rods. **(A)** SDS-PAGE (Coomassie staining) of a representative purification of septin octamers containing SEPT9_FL_ and SEPT9_G568_. Fractions from IMAC and SEC including the major and minor peak are indicated. SEPT9 degradation products are marked with arrows. The anti-SEPT9 Western blot shows the SEPT9 degradation products. **(B)** Representative MS analysis of the purified septin complexes. Both the main product peak and the minor peak from SEC were analyzed. Peptide abundancies of the SEPT_FL_ (left panel) and SEPT9_G568_ (right panel) octamer are plotted; the indicated intensity score reflects the accumulated intensities of all peptides detected for the respective subunit. **(C)** Nucleotide content of octameric rods containing SEPT9_FL_ or SEPT9_G568_ detected by analytical anion exchange chromatography (representative runs out of at least three per rod species performed) after heat denaturation of the protein. Calibration runs with GTP and GDP are shown in the upper row.

SDS-PAGE and Western blot analysis with an anti-SEPT9 antibody documented the presence of shorter fragments of SEPT9 (Figure 1A). SEPT9 has two flexible regions, a long extension at the N-terminus and a shorter one at the C-terminus. We suspected one or both of these extensions to cause the shorter fragments. The long SEPT9 isoforms used by Iv et al. (2021) in their rod preparations contained both the N-terminus or larger parts thereof, but their SEPT9i3 terminates at residue Glycine 568. This residue represents the terminal residue of the α6 helix of the G domain. We decided to test first if a version of SEPT9 truncated at Glycine 568 (SEPT9_G568_) still produces degradation products in our purification pipeline. Octameric rods expressing SEPT9_G568_ showed less degradation products than the native octamer and the purified complex could be readily separated from other subcomplexes and contaminants by the preparative SEC (Figure 1A). The IMAC enriched septin preparation was analyzed on a glycerol gradient centrifugation to confirm the integrity of the complex. Septin subunits, SEPT9 degradation products and contaminants migrated in the fractions corresponding to a lower molecular weight whereas the stoichiometric combination of all four subunits migrated just before the 670 kD marker protein became abundant, indicative of an octameric septin complex (Supplementary Figure S1B).

We analyzed the fractions from the major and minor peak from the SEC of SEPT9_FL_ and SEPT9_G568_ complexes by mass spectrometry (MS). All four subunits are present in nearly stoichiometric abundance (Figure 1B). We conclude that our septin preparation in the main peak is indeed an octameric complex containing all four subunits. We performed the MS analyses with several purified complexes. The peptide intensity scores of the individual subunits varied slightly between the preparations, but we observed in all MS runs that SEPT9 was less abundant in the complex of the minor SEC peak. As this subcomplex poorly formed filaments (see below), all subsequent experiments were conducted with a septin preparation from the main peak.

We subsequently determined the nucleotide content of our rod preparations. Bound nucleotide was released from the protein by heat denaturation and afterwards analyzed by analytical anion exchange chromatography (Figure 1C). We determined the peak integrals and calculated the ratio of GDP to GTP. Both SEPT9_FL_ and SEPT9_G568_ complexes contained between 3.5 and 5 times more GDP than GTP (varying between several purifications performed). Overall, we could not detect any difference in SEPT9 and SEPT9_G568_ containing octamers regarding the nucleotide content after purification.

Hexameric SEPT2/6/7 complexes were purified using the same purification pipeline and yielded a highly purified after SEC (Supplementary Figure 1C).

### 3.2 Filament Forming Properties

**FIGURE 2:**
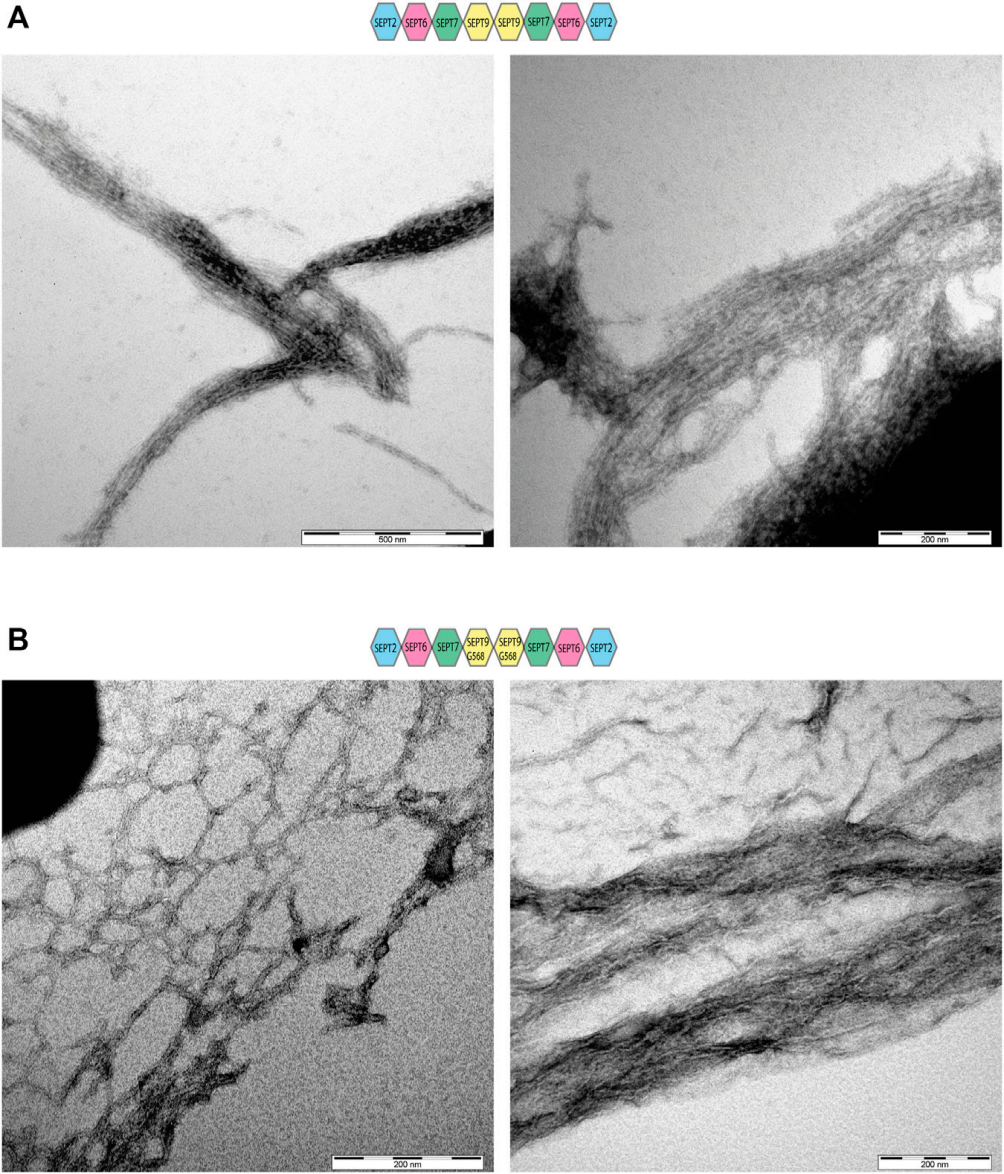
Electron microscopy of SEPT9_FL_ and SEPT9_G568_ containing septin rods. Negative stains of septin filaments obtained by dialysis in low salt buffer showing bundles and network-like structures. **(A)** Electron microscopy of septin filaments containing SEPT9_FL_. **(B)** Electron microscopy of septin filaments containing SEPT9_G568_.

We asked next whether septin filament formation is altered by the absence of the SEPT9 C-terminal extension. Septin preparations were cleared from aggregates by centrifugation, dialyzed into low salt buffer and subjected subsequently to ultracentrifugation. Surprisingly, we could detect filaments already at 300 mM NaCl for SEPT9_FL_ octamers (Supplementary Figure S2A) whereas filaments of SEPT9_G568_ containing octamers and hexamers required 50 mM NaCl to form (Supplementary Figures S2B, C). Filament formation was almost abolished at 100 mM NaCl. Negative stain EM of SEPT9_FL_ octamers revealed the presence of long filament bundles consisting of parallel aligned septin filaments, consistent with the findings of Iv et al. (2021) (Figure 2A). SEPT9_G568_ containing filaments formed also bundles, but the filaments forming the bundle appeared rather curved and less parallel aligned (Figure 2B). Complexes isolated from the minor peaks of the SEC only poorly formed filaments (data not shown) which we were not able to visualize by EM.

### 3.3 Effects of Human Septin Rods on the Polymerization Properties of Actin

The septin subunit SEPT9 was reported to bundle actin and microtubules (Bai et al., 2013; Dolat et al., 2014; Mavrakis et al., 2014; Smith et al., 2015).

**FIGURE 3:**
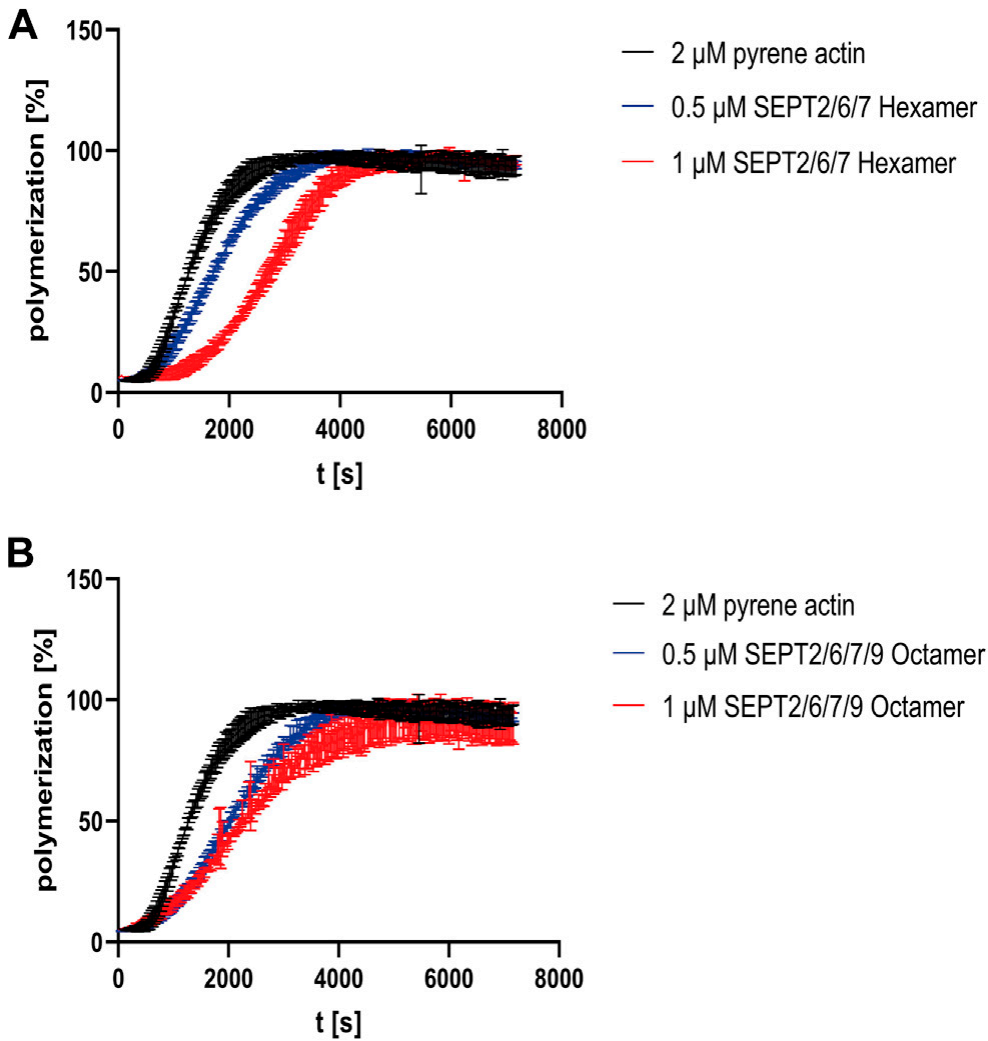
Influence of human septin rods on actin polymerization. **(A)** Actin polymerization assay using pyrene actin with and without septin hexamers. **(B)** Actin polymerization assay using pyrene actin with and without SEPT9_G568_ containing octamers. The assays shown were performed in triplicate and in quintuplicate for actin. The same averaged actin curve was used for evaluation of the assay. The unprocessed raw data are shown in Supplementary Figure S4.

To investigate the influence of septin rods on the formation of the actin cytoskeleton *in vitro*, we performed actin polymerization assays with 2 µM pyrene labeled G-actin and recorded the increase of fluorescence over time. G actin without additives or with purified GST (serving as control to exclude unwanted side effects by crowding) displayed a typical sigmoidal polymerization curve (Supplementary Figure S3A) (Cooper et al., 1983) which can be described mathematically by a growth curve including lag phase, exponential growth and plateau phase. From this curve, parameters such as the rate constant, the lag time or the doubling time can be calculated. All parameters calculated from the polymerization curves are summarized in Supplementary Table S1. Actin polymerization did not occur in G-buffer without salts (Supplementary Figure S3B). The presence of hexameric septins increased the lag time in a concentration dependent manner, leading to a shift of the inflection point relative to the inflection point of the actin curve (Figure 3A). For octamers, the shift was more pronounced at lower concentration but did not change significantly at higher septin concentrations (Figure 3B). More important, the actin monomer turnover in the elongation phase (represented by *τ*, the inverse value of the rate constant) was slightly decreased about 1.4 fold for hexameric rods and 0.5 µM octamer relative to actin alone and more pronounced decreased by 2.1 fold for 1 µM octamer. Increased *τ* values are reflected by a shallower slope of the curve.

A spin down assay with septin octamers previously dialyzed in G buffer showed that septin filaments did not measurably bind G-actin (Supplementary Figure 3C).

### 3.4 Nucleotide Uptake and Hydrolysis Properties of the Human Septins

**FIGURE 4:**
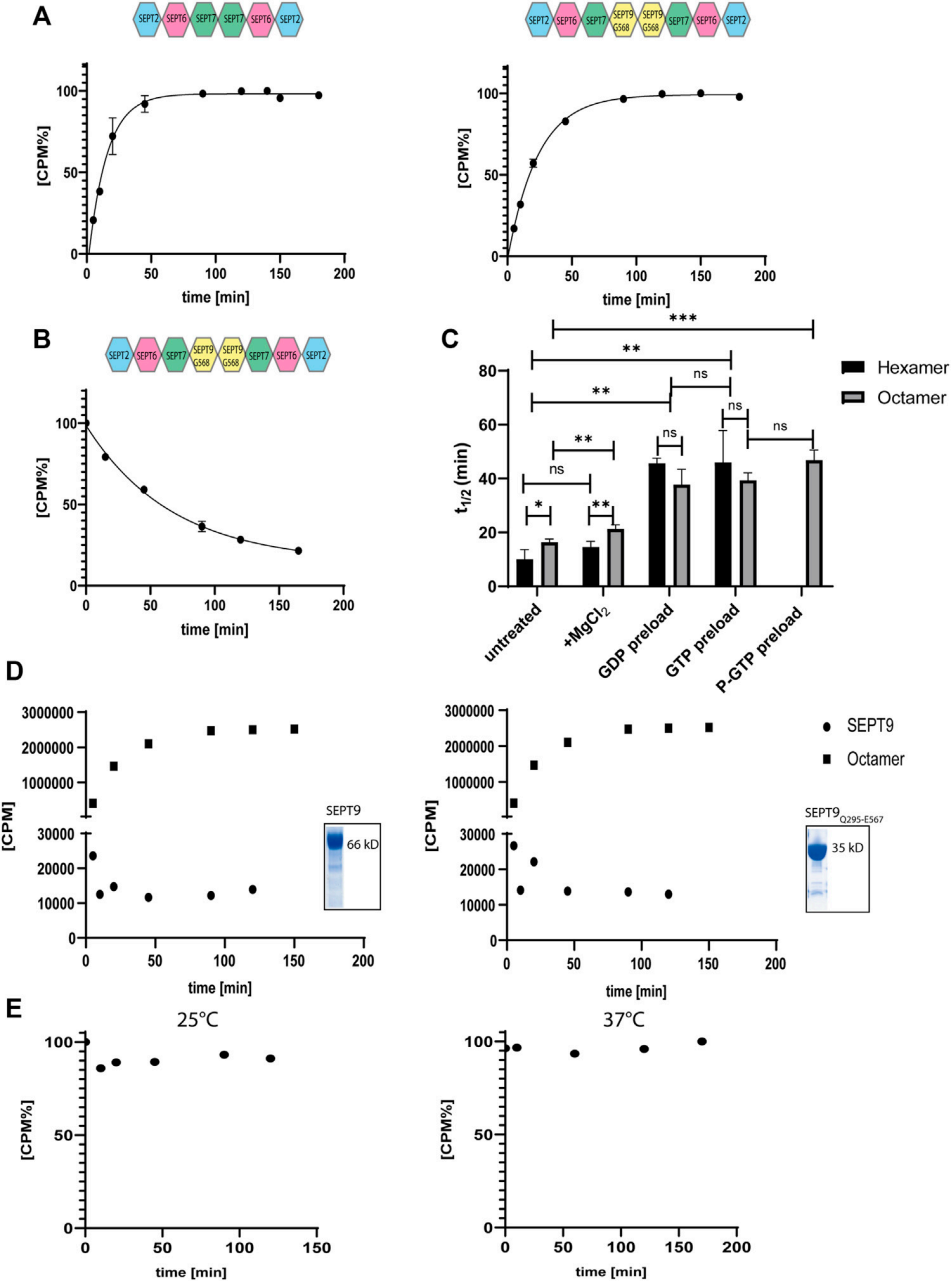
Nucleotide uptake- and hydrolysis properties of hexameric and SEPT9_G568_ octameric septin rods. **(A)** Nucleotide exchange reaction of hexameric (left panel) and octameric septins (right panel). Purified complexes were incubated with [γ^32^P]-GTP and uptake was monitored at the indicated timepoints by a filter assay. [CPM%] values (normalized radioactive counts) are plotted vs. the reaction time. The connecting line represents the fitting curve of the exponential association. **(B)** Nucleotide exchange reaction of octameric septins preloaded with [γ^32^P]-GTP. The preloaded complex was incubated with GTP and decrease of [γ^32^P]-GTP was monitored at the indicated timepoints by a filter assay as in A. The connecting line represents the fitting curve of the exponential dissociation. **(C)** Compilation of the t_1/2_ values of all performed nucleotide exchange assays. The parameters of the statistical evaluation are provided in Supplementary Tables S2, S3. Error bars represent the standard deviation. All assays [including those depicted in detail in (A,B)] were performed at least in triplicate. **(D)** Representative [γ^32^P]-GTP uptake assay for isolated SEPT9_FL_ (left panel) and SEPT9_Q295-E567_ (right panel) subunits. The assay was performed as in (A). The raw, unprocessed CPM counts are plotted vs. the reaction time. For comparison, a representative nucleotide uptake reaction for a septin octamer is also plotted. The inlets show the Coomassie stained SDS-PAGE of both purifications. **(E)** Representative GTP hydrolysis assay for octameric septins. After preloading with [γ^32^P]-GTP, the complex was subjected to hydrolysis conditions applicable for small GTPases. The reaction was performed at 25°C (left panel) and 37°C (right panel). Samples were taken at the indicated time points and the [γ^32^P]-GTP content was detected by a filter assay. [CPM%] values (normalized radioactive counts) are plotted vs. the reaction time. GTP hydroloysis of hexameric rods is shown in Supplementary Figure S4.

We have previously measured the uptake of [γ^32^P] labelled GTP in the presence of EDTA by the hexameric- and octameric septin rods from yeast (Baur et al., 2018). All following experiments were conducted with SEPT9_G568_ containing octamers. [γ^32^P]-GTP uptake followed an exponential one phase association kinetics from which the rate constant and the reaction half time t_1/2_ can be calculated. The reaction half time t_1/2_ was 10 min for hexameric rods and 16 min for octameric rods, respectively (Figure 4A). All t_1/2_ values of this and the subsequently performed experiments are summarized in Figure 4C and the full evaluation of all performed measurements are shown in the supporting information (Supplementary Tables S2–S4).

Unlike for yeast septin rods, adding MgCl_2_ to the exchange reaction after 20 min did not change the overall shape of the uptake kinetics but prolonged t_1/2_ slightly (and barely significantly) to 14.5 min for hexamers and 21 min for octamers (Supplementary Figure S5A and Figure 4C).

To evaluate whether the bound nucleotide remains in the septin rod or exchanges with free GTP, we pre-loaded septin preparations with non-radioactive GDP or GTP and removed excess nucleotide by micro-dialysis. Subsequent nucleotide exchange against [γ^32^P]-GTP showed a shift in reaction half times to 42 min for both GDP and GTP loaded hexameric rods and to 33 min for GDP loaded octameric rods and 38 min for GTP loaded octameric rods, respectively (Supplementary Figures S5B, C and Figure 4C).

We also subjected the octameric rods to exchange of previously bound [γ^32^P]-GTP against non-radioactive nucleotide. After loading, excess nucleotide was removed via a desalting column and the uptake of non-radioactive GTP was monitored via the decrease of protein-bound radioactivity. The calculated reaction half time of 46.5 min was slightly (and not significantly) slower than the t_1/2_ for the inverse exchange reaction (GTP against [γ^32^P]-GTP) (Figure 4B).

We conclude that human septin octamers exchange bound nucleotides quite readily.

Previous studies showed that isolated septin subunits may have other GTPase properties than septin rods (Sheffield et al., 2003). We tested the isolated SEPT9_FL_ subunit and the SEPT9 G-domain (SEPT9_Q295-E567_) for nucleotide uptake and found that both did not accept [γ^32^P]-GTP (Figure 4D).

To find out whether human septins follow the same GTP hydrolysis mechanism like other small GTPases, we measured the released or protein-bound radioactivity by [γ^32^P]-GTP preloaded septins under conditions used for other small GTPases (i.e. magnesium and an excess of non-radioactive GTP to avoid unwanted re-association of [γ^32^P]-GTP molecules) (Tucker et al., 1986; Schweins et al., 1995). In contrast to common small GTPases of the Ras family, neither hexameric nor octameric rods showed a release of hydrolyzed [γ^32^P] under these conditions in the applied time frame, regardless of the applied reaction temperature (25°C or 37°C, respectively) (Figure 4E and Supplementary Figure S6).

## 4 Discussion

### 4.1 The C-Terminus of SEPT9 Contributes to Filament Formation

Human hexameric septin rods are available for *in vitro* experimentation and even crystallization for several years (Sheffield et al., 2003; Sirajuddin et al., 2007). However, purified octameric rods containing SEPT9 were only very recently obtained (Iv et al., 2021). We introduce here an additional two step purification protocol for human octameric rods harboring a hexa-histidine tag at only one of its subunits. Gycerol density gradient centrifugation of the complex after the first purification step separated contaminants and septin containing subcomplexes from the octamer, which migrated at the expected density in the gradient. After preparative SEC, we obtained the purified octamer including all four subunits in nearly stoichiometric abundance as judged by MS analysis.

The central subunit SEPT9 is prone to C-terminal degradation during expression and purification of the rod. Removal of the terminal 18 amino acids stabilized the protein and led to less degradation products. To our surprise we found that the SEPT9_FL_ containing rods formed filaments already at 300 mM NaCl whereas filament formation by hexamers and SEPT9_G568_ containing octamers was abolished already at 100 mM NaCl. The first study of filament formation of SEPT2/6/7 hexamers raised the expectation of another—at that time largely unknown - septin subunit (SEPT9) with a stabilizing effect on the filaments (Sheffield et al., 2003). Based on our herein presented experimental data we propose that the stabilizing effect of the SEPT9 subunit on filament formation is mediated by the α6 helix and its C-terminal extension. The α6 helix of the SEPT9 G domain is an essential component of the central SEPT9-SEPT9 NC interface, contributing to an interaction network with the α2 helix of the neighboring subunit (Castro et al., 2020). The C-terminal extensions would point out from the interface, maybe interacting with each other they further stabile the interface. We and others (Iv et al., 2021) postulate that the assembly of higher ordered septin structures is driven *in vitro* by the N- and C-terminal extensions.

### 4.2 Human Septin Complexes Reduce the Rate of Actin Polymerization *in vitro*

Several studies have already demonstrated the capability of SEPT9, septin hexamers and octamers to bind and bundle filamentous actin (Mavrakis et al., 2014; Smith et al., 2015; Iv et al., 2021). All these studies monitored the effects of septins on filamentous actin. Actin bundling was detected subsequently by electron- or fluorescence microscopy. The pyrene actin assay introduced in this study looks at actin polymerization in real time, however, it measures the net polymer weight and does not provide information on the longitudinal distribution of the filaments (Cooper et al., 1983). It was recently shown by *in vitro* microscopy that recombinant septin rods cross-link actin filaments into straight and curved bundles (Iv et al., 2021). Our assay revealed that the presence of septin rods decreases the rate of actin polymerization. As we could not detect any interaction between septin rods and G-actin we propose that the septins exert their influence on polymerization through interaction with the forming actin filament and subsequently bundle filaments by lateral joining. Why this effect is more pronounced for octamers requires further investigation. Maybe the interaction of the octamer with the actin filament is favored over hexamer binding.

Whether SEPT9 is a bundling factor on its own is currently under debate (Mavrakis et al., 2014; Smith et al., 2015; Iv et al., 2021). As the influence on actin monomer turnover is more pronounced for octamers, we support a the idea of a direct contribution of SEPT9 on actin polymerization.

### 4.3 Nucleotide Uptake- and Hydrolysis by Human Septin Complexes

Septins were first labeled as GTP binding proteins in Drosophila (Field et al., 1996) and afterwards nucleotide binding was confirmed also for human and yeast septin complexes (Sheffield et al., 2003; Vrabioiu et al., 2004) and subsequently evaluated in further studies (Farkasovsky et al., 2005; Baur et al., 2018). However, the functional role of nucleotide binding- and hydrolysis of the septins in cellular processes and in septin (proto-)filament assembly is still under debate and not well understood.

The availability of yeast and human septin octamers enabled us to measure nucleotide association kinetics in these protein complexes and to compare the properties of both species. GTP-loading in human septin rods remains on a constant level after reaching a plateau whereas in the yeast septin rods the radioactive signal decreases about 50% after reaching a maximum (Baur et al., 2018). This indicates the release of the γ-phosphate or the entire nucleotide in at least some subunits of the protofilaments. Mg^2+^ suppresses this release but shows no significant effect on the GTP association rate in the yeast septin rods. In contrast, Mg^2+^ slightly slows down the GTP binding in human septin rods. In the absence of Mg^2+^, hexameric and octameric rods from both species are able to exchange previously bound GTP and GDP. The exchange rates for GDP and GTP of the human septin rods do not differ significantly. In contrast, the *S. cerevisiae* septin rods exhibit a much faster GDP-GTP than GTP-GTP exchange. These discrepancies indicate that, although displaying a high structural similarity, the septins from different species follow each an individual biochemistry. Even within one species the different subunits exhibit different properties. Comparing the GTP and GDP loaded structures of SEPT2 (PDB IDs 3FTQ and 2QNR, respectively) reveals a conformational change in the Switch1 region with the invariant Thr78 and Mg^2+^ being in close contact with the γ-phosphate exclusively in the GTP conformation. In the GDP conformation Switch1 is either unstructured or oriented away from the nucleotide (Supplementary Figure S7A). This conformational change cannot be seen in the structures of SEPT9 (PDB IDs 5CYP and 5CYO) where the corresponding Thr64 in Switch1 is associated with Mg^2+^ towards the nucleotide in both nucleotide states (Supplementary Figure S7B). This inflexibility of Switch1 might explain the inability of the isolated SEPT9 to accept [γ^32^P]-GTP and why we do not detect significant differences in nucleotide uptake between hexamers and octamers. However, it should be kept in mind that the mentioned structures do not contain a native G interface and thus they can provide only indications explaining our experimental findings.

GTP hydrolysis is routinely detected in small GTPases of the Ras family. Experimentally, the small GTPase is loaded with GTP in the presence of EDTA and then subjected to a Mg^2+^ containing buffer without EDTA. The Mg^2+^ ion is an indispensable component for the GTPase reaction. The γ-phosphate is coordinated by invariant threonine and glycine residues in the Switch1 and Switch2 regions. The conformational change following the GTPase reaction was termed “loaded-spring mechanism” which allows the release of the γ-phosphate after hydrolysis and the relaxation of the switch regions into the GDP conformation (Vetter and Wittinghofer 2001). We were not able to detect the release of [γ^32^P]-GTP or hydrolyzed [γ^32^P] from any rod species examined. This indicates that bound nucleotides and presumable hydrolysis products remain in the binding pocket and are not released into the surrounding medium, at least not in the timeframe of the experiment (120–180 min) and at least not under the conditions applied. This is more in line with our findings for yeast septins (Baur et al., 2018) rather than with the properties of small GTPases of the Ras family.

GTPase hydrolysis products were detected in yeast and mammalian septin preparations after denaturation of the protein (Sheffield et al., 2003; Versele and Thorner 2004; Sirajuddin et al., 2009; Castro et al., 2020). In contrast, our assay was conducted under native conditions. Consequently, hydrolysis occurs either at very slow pace, undetectable within the chosen timeframe, or the hydrolysis products remain in the active site after the reaction took place. This raises the question which role nucleotides and their hydrolysis play. This question was addressed in yeast septins and it was proposed that nucleotide hydrolysis plays a role during formation of the rod (Weems and McMurray 2017). This would indeed imply that one GTP hydrolysis event is enough and that cycling between the GDP and GTP bound state would not be necessary. However, more studies both *in vivo* and *in vitro* are necessary to convincingly address this issue for human septins.

Taken together, we propose that GTP binding and hydrolysis in septins follow a different mechanism than in other small GTPases and that septin rods from budding yeast differ mechanistically in their guanine nucleotide interactions from their human counterparts. More structural information from yeast septins is needed to understand these differences.

## Data Availability

The original contributions presented in the study are included in the article/Supplementary Material, further inquiries can be directed to the corresponding author.
