# Supplementary material for: Biochemical Characterization of a Human Septin Octamer
Source: Front Cell Dev Biol. 2022 Mar 3;10:771388. doi: 10.3389/fcell.2022.771388 (PMC8928218; doi:10.3389/fcell.2022.771388)

Supporting Information: Supplementary Tables and Figures.

Supplementary Table 1

Evaluation of the actin polymerization reaction. All measurements were performed in triplicate. Y0 is the start value at t=0, Y_M is the maximum value, the rate constant K equals the velocity of actin polymerization, τ describes the mean doubling time of polymerizing actin and the doubling time $t_{1/2}$ represents the time span after which the initial fluorescence has doubled its value. t_{ip} represents the time point of the inflection point and can be used to compare the time shifts of the individual curves on the x axis.

	2 µM actin	+ 0.5 µM hexamer	+ 1 µM hexamer	+ 0.5 µM octamer	+ 1µM octamer
Y0	5.77	7.32	8.46	8.38	8.65
Y_M	96.77	98.28	98.96	97.19	94.50
K [s^{-1}]	0.001758	0.001291	0.001262	0.001264	0.000833
τ [s]	569	775	792	791	1200
$t_{1/2}$ [s]	394.4	536.9	549.2	548.5	832.1
t_{ip} [s]	1137.1	1518.0	2547.7	1816.8	1861.6
R^2 (fit)	0.9368	0.9703	0.9476	0.9508	0.8753

Supplementary Table 2

Summary of the performed fits for the nucleotide uptake measurements as shown in the figures. $t_{1/2}$ values were calculated as mean from the respective one phase exponential fit. All measurements were performed in triplicate.

Septin construct	Preload nucleotide	K (min^{-1})	$t_{1/2}$ (min)	95 % CI of $t_{1/2}$	R^2 (fit)
Octamer	-	0,04225	16,41	15,18 to 17,75	0,9974
Hexamer	-	0,06916	10,02	7,850 to 12,77	0,9759
Octamer + $MgCl_2$	-	0,03293	21.05	18,04 to 24,70	0,9916
Hexamer + $MgCl_2$	-	0,04794	14,46	12,32 to 17,02	0,9898
Octamer	GDP	0,02076	33,39	25,96 to 45,06	0,9854
Octamer	GTP	0,01823	38,01	32,56 to 45,25	0,9953
Octamer	^{32}P-GTP	0,0149	46,58	40,54 to 54,20	0,9957
Hexamer	GDP	0,01604	43,21	37,37 to 50,82	0,9964
Hexamer	GTP	0,01684	41,15	29,73 to 62,65	0,9776

Supplementary Table 3

Evaluation of the nucleotide uptake data sets (each performed in triplicate) with unpaired t-tests. $t_{1/2}$ values were individually calculated for each replicate and provided as triplicate to the statistics analysis.
Pairwise comparisons of the GDP and GTP preloading experiments are only provided for completeness. ANOVA analysis is more applicable for these data.

	Mean $t_{1/2}$ octamer	Mean $t_{1/2}$ hexamer	Significance	p-value
untreated	16,37	10,05	*	0,04
+$MgCl_2$	21,31	14,66	**	0,01
GDP preload	37,79	45,67	ns	0,08
GTP preload	39,39	45,96	ns	0,40
	Mean $t_{1/2}$ untreated	Mean $t_{1/2}$ + $MgCl_2$	Significance	p-value
Hexamer	10,05	14,66	ns	0,13
Octamer	16,37	21,31	**	0,01
	Mean $t_{1/2}$ untreated (min)	Mean $t_{1/2}$ GDP preload (min)	Significance	p-value
Hexamer	10,05	45,67	***	<0,001
Octamer	16,37	37,79	**	0,003
	Mean $t_{1/2}$ untreated	Mean $t_{1/2}$ GTP preload	Significance	p-value
Hexamer	10,05	45,96	**	0,007
Octamer	16,37	39,39	***	<0,001

Supplementary Table 4

Evaluation of nucleotide uptake data for GDP and GTP preloaded constructs (each performed in triplicate) with ordinary ANOVA analysis followed by Holm-Sidak's multiple comparisons test. $t_{1/2}$ values were individually calculated for each replicate and provided as triplicate to the statistics analysis.

Hexamer	Mean $t_{1/2}$ untreated	Mean $t_{1/2}$ preload	Significance	p-value
untreated vs. GDP preload	10,05	45,67	**	0,003
untreated vs. GTP preload	10,05	45,96	**	0,003
	Mean $t_{1/2}$ GDP	Mean $t_{1/2}$ GTP	Significance	p-value
GDP vs. GTP preload	45,67	45,96	ns	0,96
Octamer	Mean $t_{1/2}$ untreated	Mean $t_{1/2}$ preload	Significance	p-value
untreated vs. GDP preload	16,37	37,79	***	<0,001
untreated vs. GTP preload	16,37	39,39	***	<0,001
untreated vs. ^{32}P-GTP preload	16,37	46,83	***	<0,001
	Mean $t_{1/2}$ GDP	Mean $t_{1/2}$ GTP	Significance	p-value
GDP vs. GTP preload	37,79	39,39	ns	0,61
	Mean $t_{1/2}$ GTP	Mean $t_{1/2}$ ^{32}P-GTP	Significance	p-value
GTP vs. ^{32}P-GTP preload	39,39	46,83	ns	0,08

Supplementary Figure 1

A. Representative chromatograms of the preparative SEC purification of septin octamers containing SEPT9$_{FL}$ (left) or SEPT9$_{G568}$ (right). The major and minor peak containing the purified septin complex are indicated by arrows. The first half of the major peak represents the void peak of the column.
B. Left panel: SDS-PAGE (Coomassie staining) of the fractions from a 15% - 30% Glycerol gradient separation of the SEPT9$_{G568}$ octamer. The kD of the corresponding molecular weight marker is indicated. Right panel: SDS-PAGE (Coomassie stain) of the d marker proteins thyroglobulin (670 kD), γ-globulin (155 kD; separating in SDS-PAGE into 50 kD and 25 kD bands), ovalbumin (44 kD) and myoglobin (17 kD) separated by an 15%-30% Glycerol gradient. Fractions are numbered; B = bottom fraction
C. SDS-PAGE (Coomassie staining) of a representative purification of a septin hexamer. IMAC and SEC fractions are indicated.

A

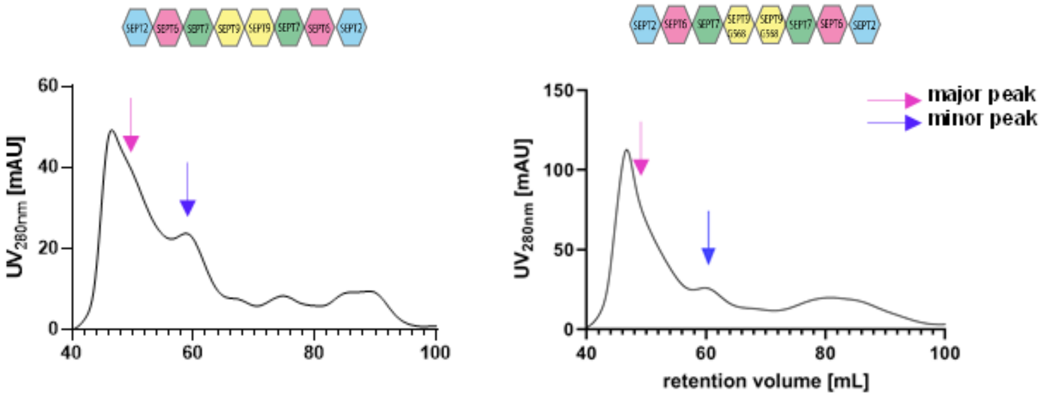

B

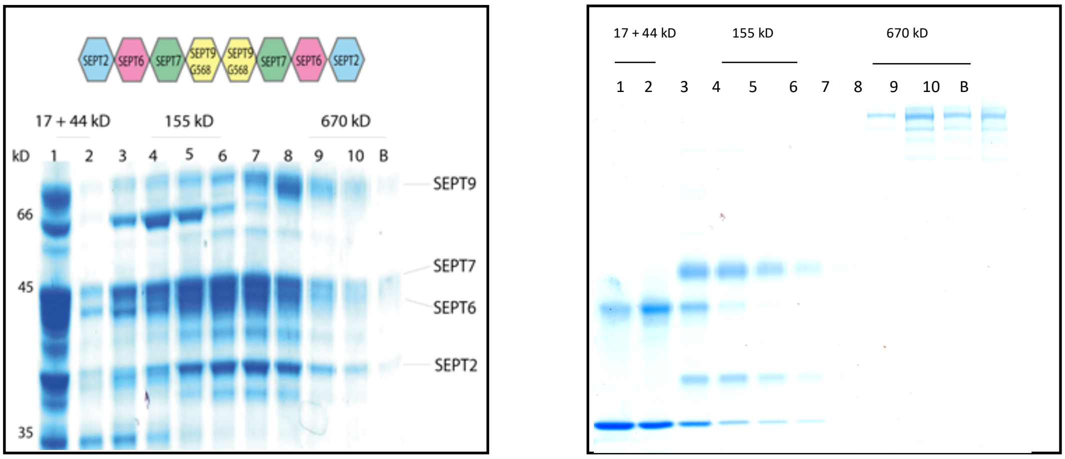

C

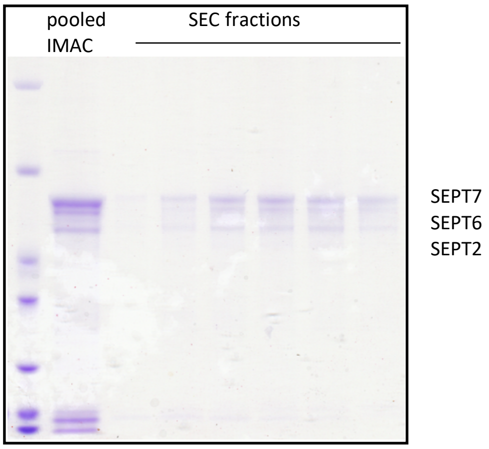

Supplementary Figure 2

A. Filament formation of SEPT9$_{FL}$ containing rods detected by a spin down assay. In SEC buffer containing 500 mM NaCl no filaments could be detected in the pellet (SEC pool). After dialysis and subsequent sedimentation, filaments could be detected in the pellet at all tested NaCl concentrations. S – supernatant, P – pellet.

B, C. Filament formation of SEPT9$_{G568}$ containing rods (B) and hexameric rods (C) detected by a spin down assay. After dialysis and subsequent sedimentation, filaments could be detected in the pellet at 0 mM and 50 mM NaCl whereas filament formation was almost abolished at 100 mM NaCl.

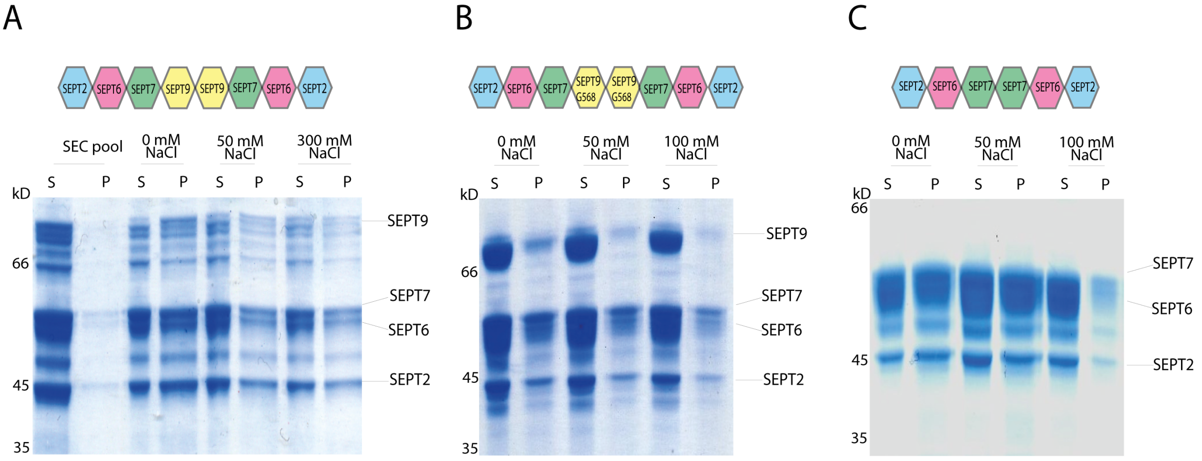

Supplementary Figure 3

A. Polymerization curve of pyren actin in polymerization buffer with and without GST.
B. Polymerization curve of pyren actin in G buffer. The assay was performed in triplicate.
C. Spin down assay of actin (A) and septin octamer (S) in G buffer. Supernatant and pellet were separated by SDS-PAGE and analyzed by Coomassie-staining. Actin was additionally detected by Western blot using a HRP coupled anti-actin antibody (lower panel). A small fraction of actin aggregates pellets under these conditions.

A

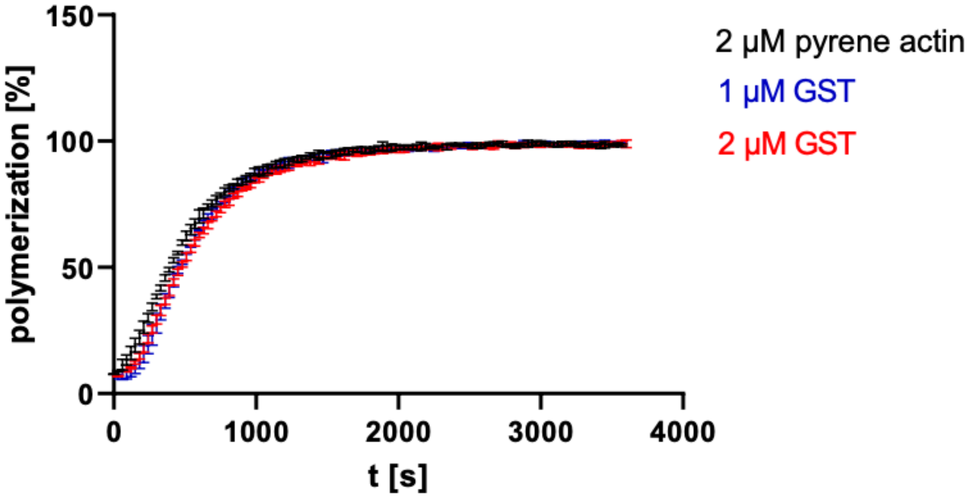

B

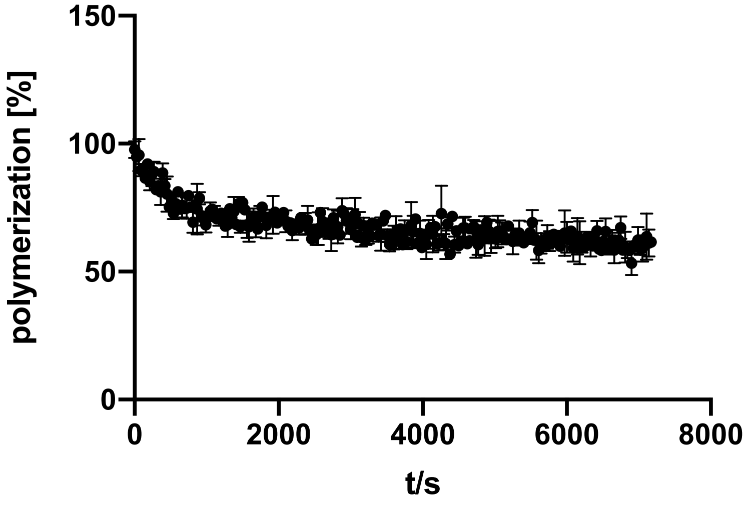

C

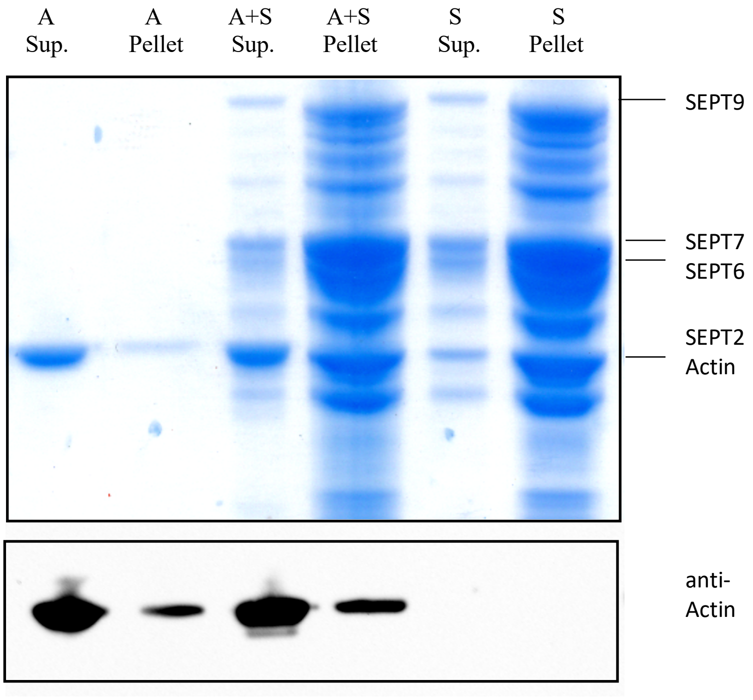
A Sup.
A Pellet
A+S Sup.
A+S Pellet
S Sup.
S Pellet
SEPT9
SEPT7
SEPT6
SEPT2
Actin
anti-Actin

Supplementary Figure 4
Unprocessed raw data to Figure 3.
The kinetics were recorded under auto-gain adjustment. In this mode the plate reader automatically chooses an initial absolute fluorescence value (usually the brightest well) and measures the intensity of the other wells in relation. Without this adjustment the brightness usually exceeds the machine's maximum detection value quickly after the lag phase, resulting in signal overflow. Consequently, the absolute values of assays are comparably only between wells of the same measurement and not directly between separate measurements. Only relative values (to the respective actin blanks) allow direct comparison between separately conducted assays.
Here, hexamer assays were conducted separately for 0.5 µM and 1.0 µM hexamer whereas octamer was assayed in one measurement. Each concentration was measured in triplicate.

A

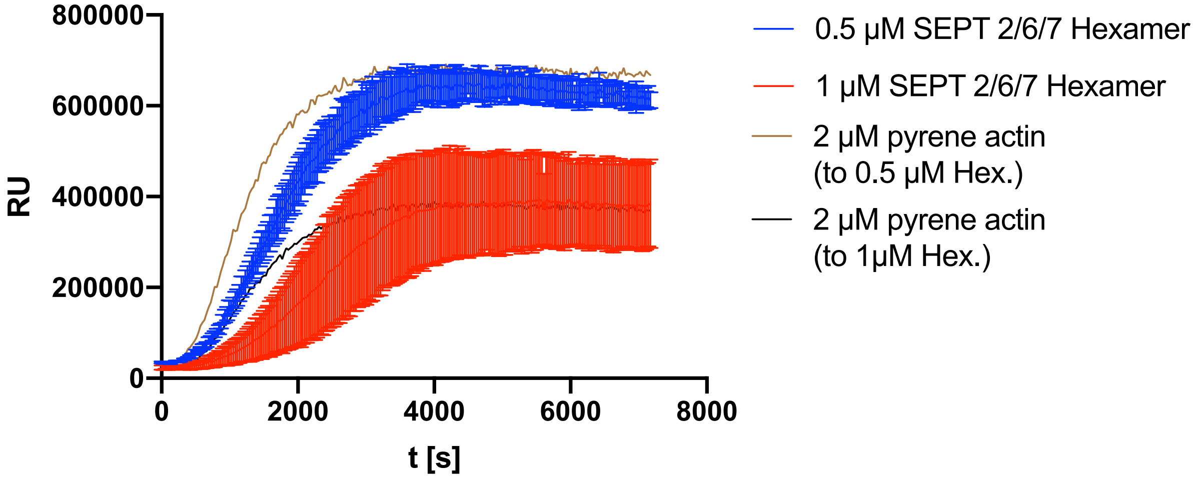

B

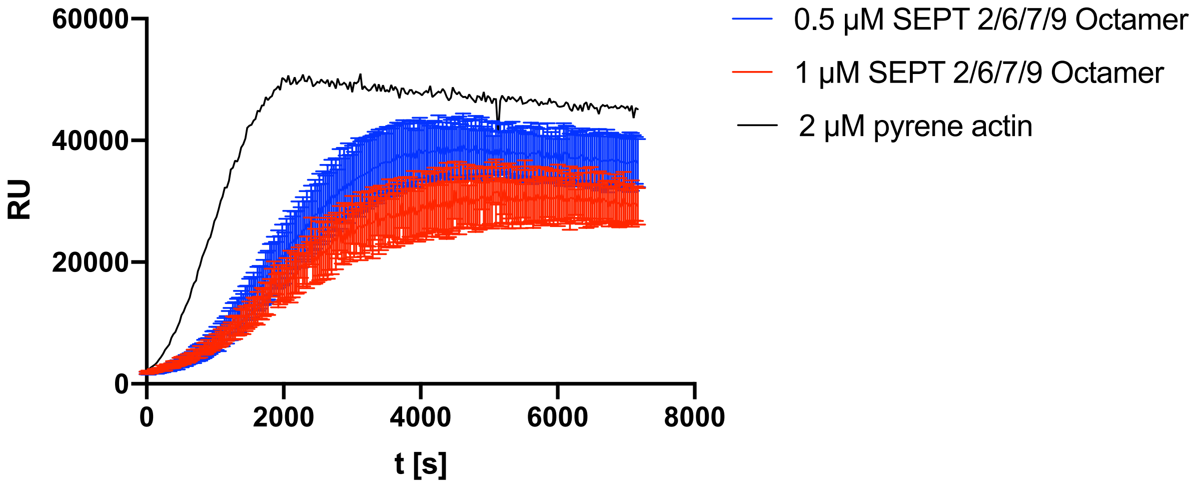

Supplementary Figure 5

Nucleotide exchange assays with various conditions. The purified septin complexes were incubated with [γ^{32}P]-GTP and uptake was monitored at the indicated timepoints by a filter assay. [CPM%] values (normalized radioactive counts) are plotted vs. the reaction time. The connecting line represents the fitting curve of the exponential association. All assays were performed at least in tripliate.

A. Nucleotide exchange reaction of hexameric (left panel) and octameric septins (right panel) with $MgCl_2$ added after 20 min.

B. Nucleotide exchange reaction of hexameric (left panel) and octameric septins (right panel) preloaded with GDP.

C. Nucleotide exchange reaction of hexameric (left panel) and octameric septins (right panel) preloaded with GTP.

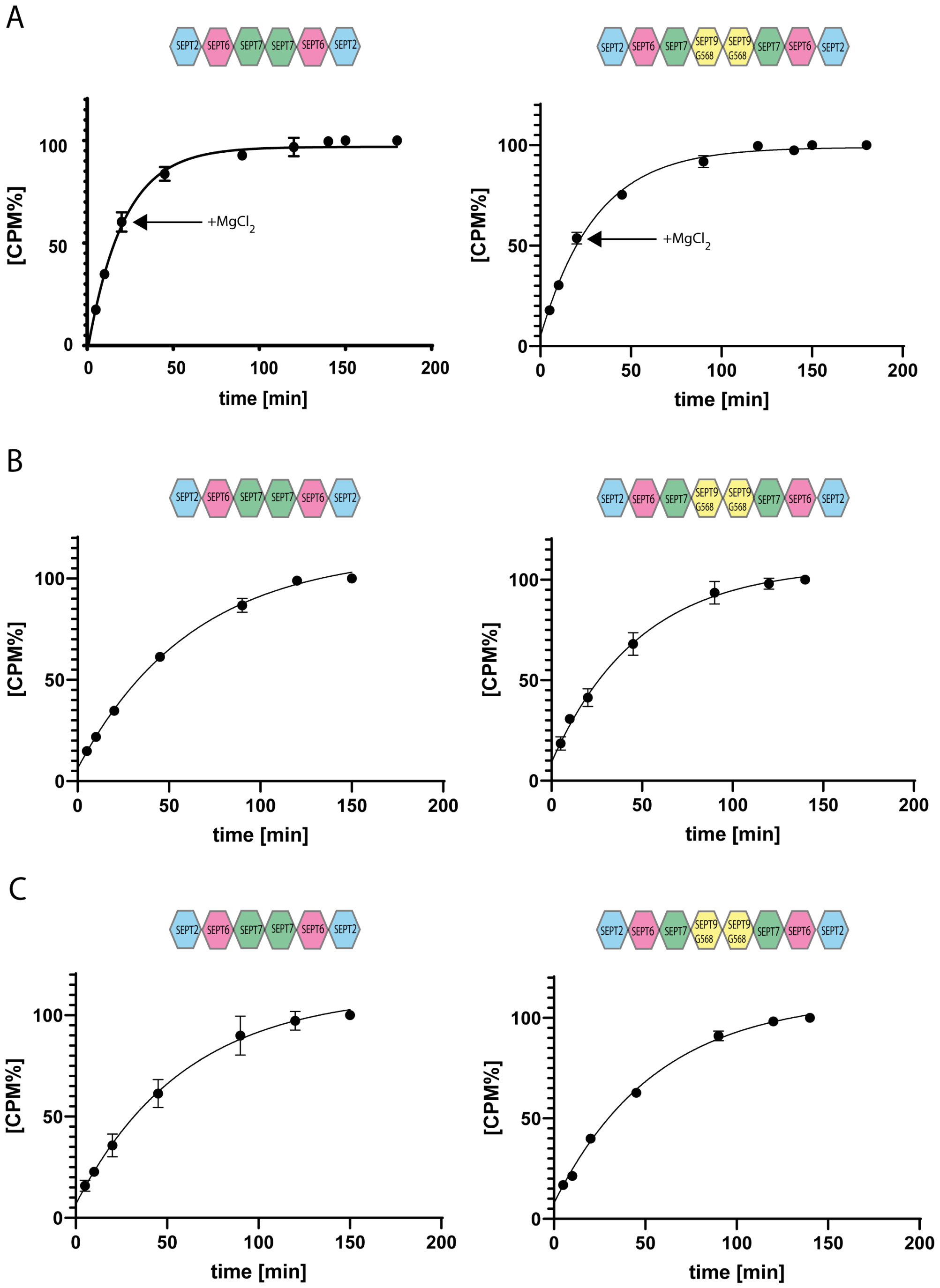

Supplementary Figure 6

Representative GTP hydrolysis assay for hexameric septins. After preloading with [γ^{32}P]-GTP, the complex was subjected to hydrolysis conditions applicable for small GTPases. The reaction was performed at 25°C.

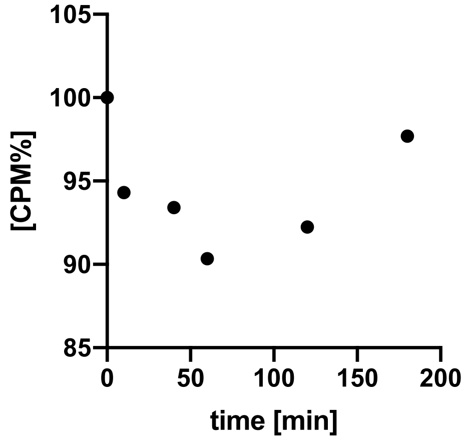

Supplementary Figure 7

A. Orientation of the Switch1 in GDP (cyan) and GTP (green) bound SEPT2. The nucleotides and Thr64 of Switch1 are shown in stick projection; the black arrow points towards Thr64. The Mg^{2+} ion is shown as green sphere. The figure was derived from PDB-Ids 2QNR and 3FTQ which show both a G-interface SEPT2 dimer. Note that in each structure one SEPT2 subunit has a partially disordered Switch1 and thus the two situations with an ordered Switch1 are shown separately.
B. Orientation of the Switch1 in GDP (cyan) and GTP (green) bound SEPT9. The nucleotides and Thr64 of Switch1 are shown in stick projection. The Mg^{2+} ion is shown as green sphere. Note the well superimposed Switch1 in both structures. The figure was derived from PDB-IDs 5CYO and 5CYP which show both a G-interface SEPT9 dimer.

A

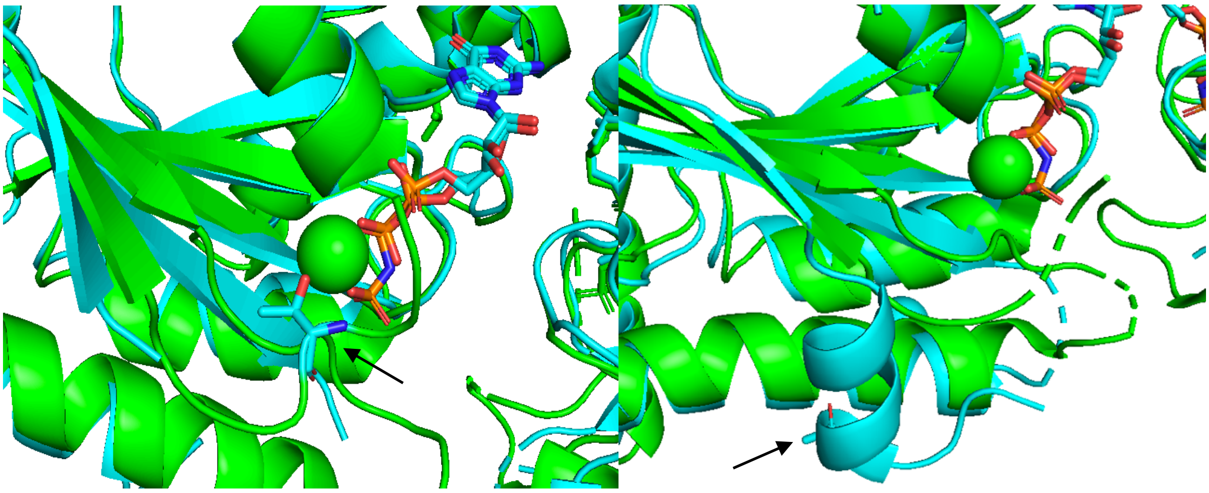

B

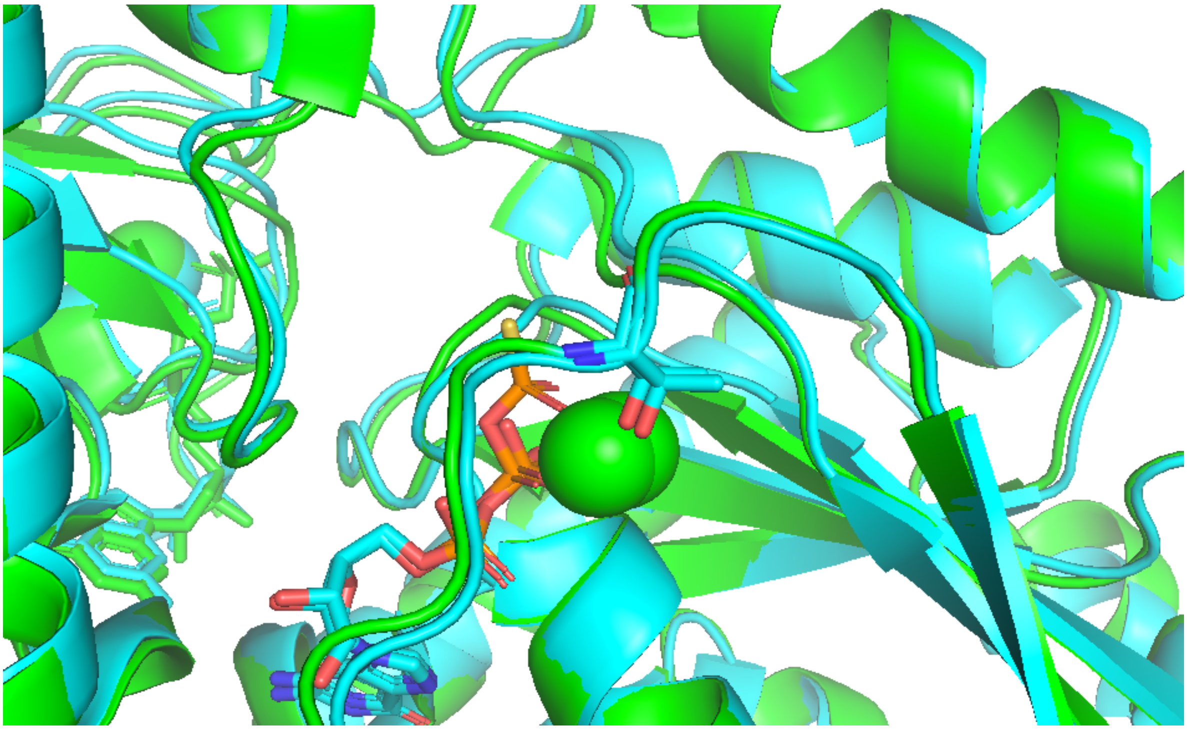

Supplement: Supplementary file 2 [file DataSheet1.PDF]
